# Algorithms of Ancestral Gene Length Reconstruction

**DOI:** 10.1155/2013/472163

**Published:** 2013-11-26

**Authors:** Alexander Bolshoy, Valery M. Kirzhner

**Affiliations:** ^1^Department of Evolutionary and Environmental Biology, Institute of Evolution, University of Haifa, 199 Aba-Hushi Avenue, Mount Carmel, Haifa 3498838, Israel; ^2^Institute of Evolution, University of Haifa, Mount Carmel., Haifa 39105, Israel; ^3^The Tauber Bioinformatics Center, University of Haifa, 199 Aba-Hushi Avenue, Mount Carmel, Haifa 3498838, Israel

## Abstract

Ancestral sequence reconstruction is a well-known problem in molecular evolution. The problem presented in this study is inspired by sequence reconstruction, but instead of leaf-associated sequences we consider only their lengths. We call this problem ancestral gene length reconstruction. It is a problem of finding an optimal labeling which minimizes the total length's sum of the edges, where both a tree and nonnegative integers associated with corresponding leaves of the tree are the input. In this paper we give a linear algorithm to solve the problem on binary trees for the Manhattan cost function *s*(*v*, *w*) = |*π*(*v*) − *π*(*w*)|.

## 1. Introduction

Ancestral sequence reconstruction (ASR) is a well-recognized problem in molecular evolution [1]. Let **G** be a (phylogenetic) tree with **n** leaf nodes, and *k* strings over one alphabet (gene sequences) assigned to *k* leaves (*k* ≤ *n*). ASR may be defined in the following way: assignment of strings to inner nodes “in the best possible way.” There are two main paradigms for ASR: maximum parsimony (MP) and probabilistic-based reconstruction. The latter includes maximum likelihood (ML) and Bayesian reconstructions. MP reconstruction has a time complexity linear in the number of sequences analyzed. The problem of the parsimonious reconstruction of ancestral states for the given tree with the given states of its leaves (the most parsimonious assignment of the labels of internal nodes for a fixed tree topology) is a well-studied problem [2–4]. Efficient algorithms have also been developed for different types of ML-based reconstructions (reviewed in [5]). ASR methods require as input both a phylogenetic tree and a set of gene sequences associated with corresponding leaves of the tree [6].

**Figure:**
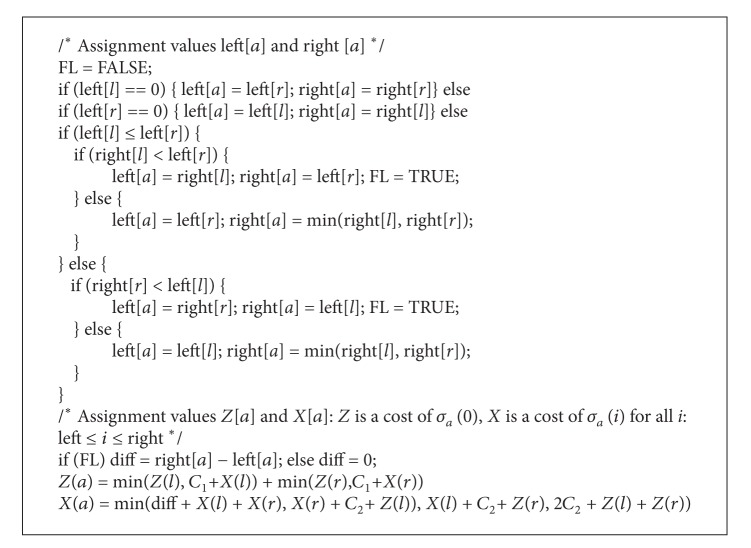
Pseudocode 1

ASR is related to gene sequence evolution while the problem presented in this paper, being inspired by ASR, deals with gene length variation. Instead of considering leaf-associated sequences we take into account only their lengths. Instead of the reconstruction of ancestral sequences, we search for the optimal reconstruction of ancestral gene lengths. The problem may be called ancestral gene length reconstruction (AGLR). AGLR is actually a problem of finding an optimal labeling which minimizes the total “length” sum of the edges, the minimum sum problem where both a tree and nonnegative integers associated with corresponding leaves of the tree are the input.

In the graph theory vertex labeling related problems were intensively studied [8]. Typically, the problems can be described as follows: for a given graph, find the optimal way of labeling the vertices with *distinct* integers. The problems and their solutions were described in [9–12]. In [13] we presented the algorithms to solve the minimum sum problem where both a tree and *positive* integers associated with *all leaves* of the tree are the input (finding the optimal way of labeling the vertices with *positive* integers). Here we would like to formulate the minimum sum problem where both a tree and *positive* integers associated with *some of the leaves* of the tree are the input (finding the optimal way of labeling the vertices with *nonnegative* integers). This problem reflects a situation in which the genome tree is constructed by one or another method for a set of genomes, the leaves of the tree are linked with the corresponding genomes of the set, and the leaves are labeled by integers designating lengths of genes of a chosen gene family. Some leaves would be labeled *zero* because corresponding genomes have no genes of the chosen gene family. Alternatively, it may be a case of a missing value but in this study we do not consider this case: in the problem definition that we bring here zero means “no value.”

In this paper we provide a linear algorithm to solve max sum problem on binary trees for the Manhattan cost function *s*(*v*, *w*) = |*π*(*v*) − *π*(*w*)|. The algorithm uses dynamic programming technique and the properties of the Manhattan distance.

## 2. Preliminaries

Let **G** be a tree with **n** leaf nodes, vertex set **V**(**G**), and edge set **E**(**G**). *N* = |*V*(*G*)|. Let us number the leaf nodes of *G*:1, 2, …, *n*. Let us number the root of *G* : *N*. An *integer labeling* ***π*** of *G* is a mapping *π* from *G* to a set of nonnegative integers, where label 0 is an out-of-the ordinary label meaning “absent value.” Let us denote integer labeling of the leaf nodes of *G*(*π*(1) = *p*_1_, …, *π*(*n*) = *p*_*n*_). Let us denote by *g*_min⁡_ and *g*_max⁡_ minimum and maximum *positive* integers labeling leaf nodes: *g*_min⁡_ = min⁡*p*_*i*_ : *p*_*i*_ > 0; *g*_max⁡_ = max⁡*p*_*i*_; *m* = *g*_max⁡_ − *g*_min⁡_ + 1.

Let us introduce a cost function *φ* of the edge *vw* ∈ *E*(*G*):

(1) $$\begin{array}{c} \varphi \left(x,y\right)=\{\begin{array}{cc} 0 & \text{if}\;x=y\;\text{else}\\ C_{1} & \text{if}\;x=0\;\text{else}\\ C_{2} & \text{if}\;y=0\;\text{else}\\ \theta \left(x,y\right), & \end{array} \end{array}$$

where the nonnegative cost function *θ*(*x*, *y*) has the following distance properties:

(i) (2) θ(x,y)≥0 x=y⟷θ(x,y)=0/∗function  is  equal  to  zero  if  and  only  if  its  arguments  are  equal  ∗/  
(ii) (3) $$\begin{array}{c} \theta \left(x,y\right)=\theta \left(y,x\right)/*\;\text{symmetry}\;*/ \end{array}$$
(iii) (4) x>y→[(θ(x,y)<θ(x,y−1)),     (θ(x,y)<θ(x+1,y))];
(iv) (5) x<y→[(θ(x,y)<θ(x−1,y)),       (θ(x,y)<θ(x,y+1))].

*C*_1_ > *C*_2_ > *m* = *g*_max⁡_ − *g*_min⁡_ + 1. *C*_1_ is a gain penalty, *C*_2_ is a loss penalty, and *θ* is a length change penalty function. Since the likelihoods of loss and gain events are likely to differ, we may need to weight them differently. This is achieved by introducing different penalties *C*_1_ > *C*_2_; the loss penalty is normally assigned a value close to *g*_max⁡_ − *g*_min⁡_, whereas the gain penalty should be larger due to biological considerations. They suggest that, on average, gene loss might be a more likely event than gene gain. Therefore, different gain penalties were used in our study similarly to as it was done in [14].

An example of a function *θ*(*x*, *y*) is |*π*(*v*)−*π*(*w*)|^*λ*^. In case of *λ* = 1 we obtain an absolute value of the difference between labelings *v* and *w*: |*π*(*v*) − *π*(*w*)|. In case of *λ* = 2 we obtain a square of the difference between labelings *v* and *w*: (*π*(*v*)−*π*(*w*))^2^.

### 2.1. An Arbitrary Tree and an Arbitrary Cost Function

Given a tree *G*, an integer labeling of the leaves of *G*(*p*_1_, …, *p*_*n*_) = 1, the gain penalty *C*_1_, the loss penalty *C*_2_, and a cost function *θ* ((1)–(5)), the minimum sum problem is to find a labeling which minimizes the total cost:

(6) $$\begin{array}{c} S\left(G\right)=\underset{\pi }{\min}\underset{\forall \{vw\}\in E\left(G\right)}{\sum }\varphi \left(\pi \left(v\right),\pi \left(w\right)\right)\;\text{over}\;\;\text{all}\;\;\pi .\; \end{array}$$

### 2.2. A Binary Tree Problem 

Given a binary tree *G*, an integer labeling of the leaves of *G*(*p*_1_, …, *p*_*n*_), the “gain” penalty *C*_1_, and the “loss” penalty *C*_2_, the *Manhattan* minimum sum problem is to find the labelings which minimize the sum *S* over all *π*

(7) S(G)=∑∀{vw}∈E(G)&π(v)≠0&π(w)≠0|π(v)−π(w)|+k1·C1+k2·C2,

where *k*_1_ is a number of edges of type (*π*(*v*) = 0 & *π*(*w*) > 0), and *k*_2_ is a number of edges of type (*π*(*v*) > 0 & *π*(*w*) = 0).

## 3. Problem Solutions

### 3.1. DP Algorithm (for the Problem (1))

Due to the properties ((2)–(5)) of the cost function *θ*(*x*, *y*) all labels of the optimal labeling must be either equal to 0 or in the interval [*g*_min⁡_, *g*_max⁡_]. As a consequence of this, the dynamic programming (DP) method is applicable for the problem. It will be easier to explain the DP method on a binary tree using *σ*_*k*_(*i*) notation. The quantity *σ*_*k*_(*i*) will be interpreted as the minimal cost, given that node *k* is assigned integer *i*, to the subtree with the node *k* as a root of the subtree.

#### 3.1.1. DP Algorithm for a Binary Tree

*Up Phase. A procedure called DP_up calculates the costs σ*_*k*_(*i*)* of all nodes V*(*G*)* of the tree G* *, given a cost function *φ*.*

When we compute *σ*_*N*_(*i*) for the root node (the index of the root is *N*), then we simply choose the minimum of these values:

(8) $$\begin{array}{c} S\left(G\right)=\underset{i}{\min}\;\sigma_{N}\left(i\right) \end{array}$$

*Initiation.* Given labeling of the leaf nodes of *G*(*p*_1_, …, *p*_*n*_} at the tips of the tree the *σ*_*i*_(*j*) are easy to compute. The cost is 0 if the observed integer *p*_*i*_ is integer *j*, and infinite otherwise.

(9) $$\begin{array}{c} \sigma_{i}\left(j\right)=\left\{\frac{\begin{array}{cc} 0 & \text{if}\;j=p_{i} \end{array}}{\begin{array}{cc} \infty & \text{otherwise} \end{array}}\right\}. \end{array}$$

*Iteration.* For the immediate common ancestor of the nodes *l* and *r*, node *a*, we have

(10) σa(0)=min⁡⁡(σl(0),C1+min⁡j⁡ σl(j))+min⁡⁡(σr(0),C1+min⁡k⁡ σr(k)),σa(i)=min⁡⁡(min⁡j⁡[θ(i,j)+σl(j)],C2+σl(0))+min⁡⁡(min⁡k⁡[θ(i,k)+σr(k)],C2+σr(0)),         ∀i,j,k∈[gmin⁡,gmax⁡].

The interpretation of this equation is immediate. The smallest possible cost given that node *a* is assigned zero is either the cost *σ*_*l*_(0) or the “gain” penalty *C*_1_ plus the minimum of *σ*_*l*_(*j*), the least of the two plus the minima of corresponding values associated with the right descendant tree. The smallest possible cost given that node *a* is assigned *i* is a sum of two values: the first one is either the cost *θ*(*x*, *y*) of the edge from node *a* to node *l*, plus the cost *σ*_*l*_(*j*) of the left descendant subtree given that node *l* is in state *j*, or the “loss” penalty *C*_2_ plus *S*_*l*_(0); the second one is the cost *θ*(*i*, *k*) of the edge from the node *a* to the node *r*, plus the cost *σ*_*r*_(*k*) of the right descendant subtree given that node *r* is in state *k*. We select those values of *j* and *k* which minimize that sum. Equation (10) is applied successively to each inner node in the tree, doing a postorder tree traversal. Finally it computes all the *σ*_*N*_(*i*), and then (8) is used to find the minimum cost for the whole tree. The complexity of the Up_phase of the algorithm is *O*(N*m*m).

*Traceback.* The procedure calculates the labels *π*(*p*) of all nodes *p* of the tree *G*.

Choose any integer *i* which provides the minimum of the *σ*_*N*_(*i*)—it is the root label. It may be either zero or a positive *i*. Doing a preorder tree traversal, successively label each inner node in the tree: for any inner node *p*, and given that a parent label *i* was reconstructed, the label *π*(*p*) = *j* is easily reconstructed as well.

#### 3.1.2. DP Algorithm for an Arbitrary Tree

*Up-Phase. A procedure DP_up calculates the costs σ*_*k*_(*i*)* of all nodes V*(*G*)* of the tree.*

Suppose that the *k*_*a*_ descendant nodes of the node *a* are called *b*_*j*_. The following equation will therefore be similar to (10) replacing the sum of *σ*_*l*_ and *σ*_*r*_ by the total sum of *σ*_*j*_1__, while *j*_1_ traverses all values of *b*_*j*_:

(11) $$\begin{array}{c} \sigma_{a}\left(0\right)=\overset{k_{a}}{\underset{j_{1}}{\sum }}\underset{}{\min}\left[\sigma_{j_{1}}\left(0\right),C_{1}+\underset{j}{\min}\;\sigma_{j_{1}}\left(j\right)\right], \end{array}$$

(12) σa(i)=∑j1kamin⁡⁡[min⁡j⁡(θ(i,j)+σj1(j)),C2+σj1(0)],          ∀i,j∈[gmin⁡,gmax⁡].

This equation is applied successively to each node in the tree, doing a postorder tree traversal. Finally it computes all the *σ*_*N*_(*i*), and then (8) is used to find the minimum cost for the whole tree.

*Down Phase.* As Traceback above: Consider the following.

### 3.2. DP Algorithm for a Manhattan Sum for a Binary Tree (Problem (2))

Manhattan distance *θ*(*π*(*v*), *π*(*w*)) is an absolute value of the difference between labelings *v* and *w* : |*π*(*v*) − *π*(*w*)|. This distance measure has the following property: if siblings have positive labels, then all integers that lie between these values may equally serve as optimal labels of a parent.

- If (*π*(*l*) ≤ *π*(*r*)), then for all *kπ*(*l*) ≤ *k* ≤ *π*(*r*) the score *θ*(*k*, *π*(*l*)) + *θ*(*k*, *π*(*r*)) = *k* − *π*(*l*) + *π*(*r*) − *k* = *π*(*r*) − *π*(*l*).
- If (*π*(*l*) ≤ *π*(*r*)), then for all *k* < *π*(*l*) ≤ *π*(*r*) the score *θ*(*k*, *π*(*l*)) + *θ*(*k*, *π*(*r*)) = *π*(*l*) − *k* + *π*(*r*) − *k* = *π*(*r*) − *π*(*l*) + 2(*π*(*l*) − *k*).
- If (*π*(*l*) ≤ *π*(*r*)), then for all *π*(*l*) ≤ *π*(*r*) < *k* the score *θ*(*k*, *π*(*l*)) + *θ*(*k*, *π*(*r*)) = *k* − *π*(*l*) + *k* − *π*(*r*) = *π*(*r*) − *π*(*l*) + 2(*k* − *π*(*r*)).

So, as it would be proven below, at the bottom-up stage of the DP algorithm it would be sufficient to assign to each node *a* in the tree *G* four values: left(*a*), right(*a*), *Z*(*a*), and *X*(*a*). The meanings of the values are as follows: left and right are bounds of an interval associated with the node *a*, *Z* is a cost value *σ*_*a*_(0), and *X* is a cost *σ*_*a*_(*i*) for any integer *i* from the interval: left ≤ *i* ≤ right. 

*Initiation.* Given labeling of the leaf nodes of *G*(*p*_1_, …, *p*_*n*_) = 1 these four values are easy to compute for the leaf nodes:

 for (*i* = 1; *i* ≤ *n*; *i* + +) if (*p*[*i*] = = 0) {*Z*[*i*] = 0; left[*i*] = 0; right[*i*] = 0; *X*[*i*] = *C*_1_ + *C*_2_} else {*Z*[*i*] = *C*_1_ + *C*_2_; left[*i*] = *p*[*i*]; right[*i*] = *p*[*i*]; *X*[*i*] = 0}.

#### 3.2.1. Examples

**Figure 1:**
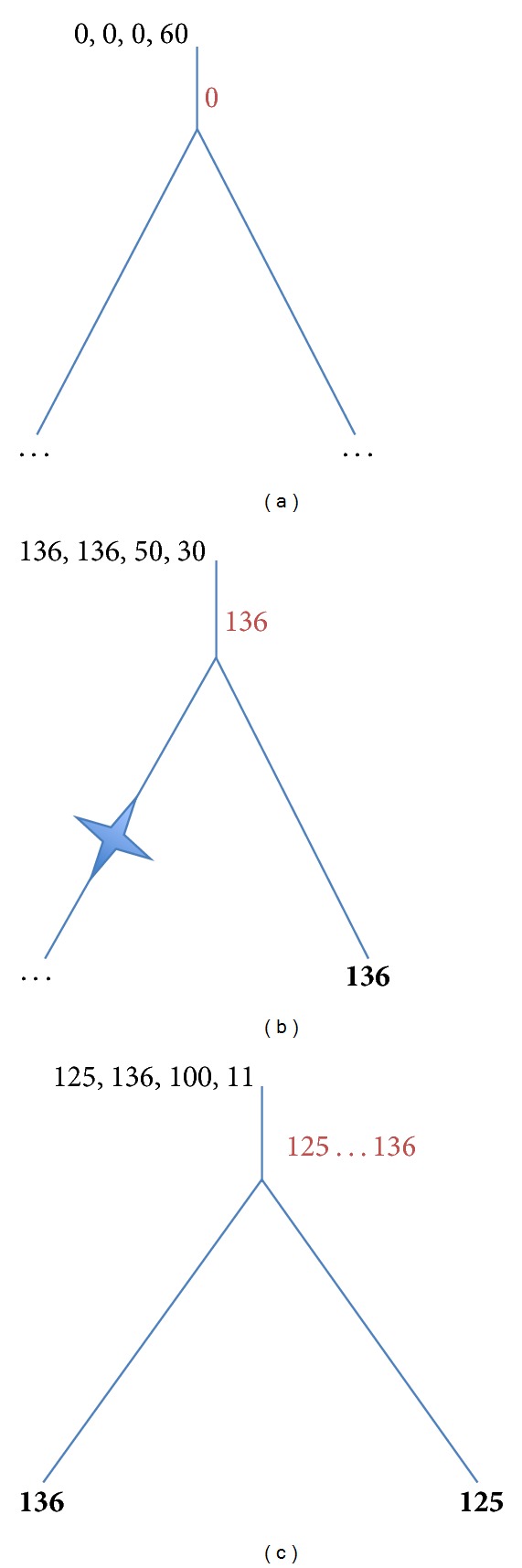
Assignment of bottom-up stage values (left, right, *Z*, and *X*) in 2-leaf trees. The “gain” penalty *C*_1_ = 50; the “loss” penalty *C*_2_ = 30. Optimal labels are in red.

Let us consider the simplest trees with two, three, and four labeled leaves. The simplest tree configuration is presented in Figure 1. There is only one node to label—the root node.

- Figure 1(a): no genes are assigned to the leaves → no gene is assigned to the root.
- Figure 1(b): the left leaf has no gene, and the right leaf has a gene with the length equal to 136 → the root is labeled by 136; the score is equal to the loss penalty *C*_2_ = 30.
- Figure 1(c): any label 125 ≤ *k* ≤ 136 is good to label the root; the score is equal to 136 − 125 = 11.

**Figure 2:**
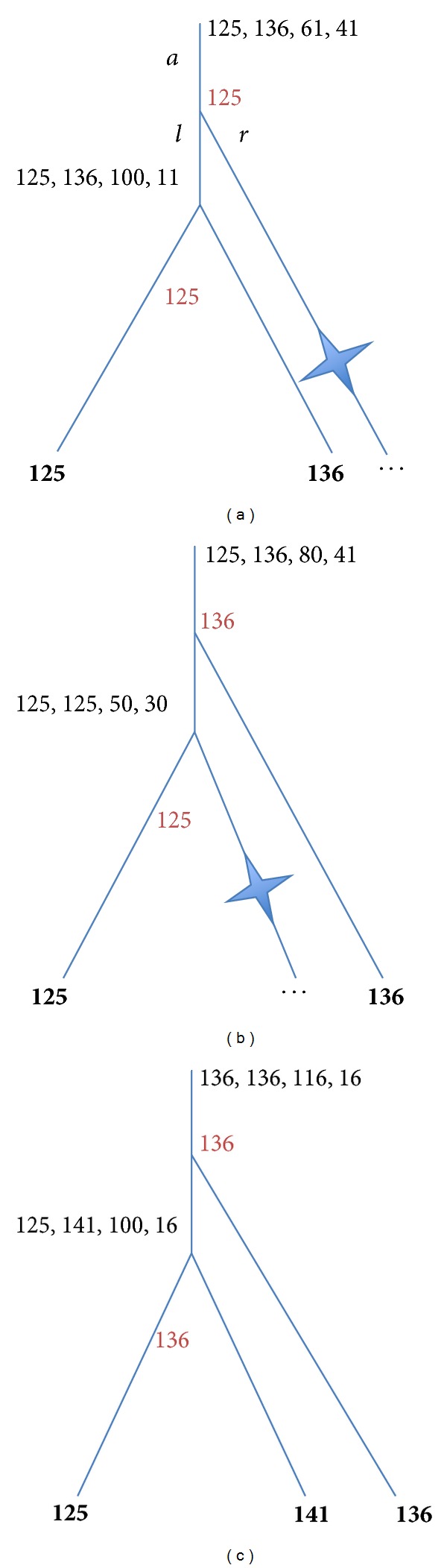
Assignment of bottom-up stage values (left, right, *Z*, and *X*) in 3-leaf trees. The “gain” penalty *C*_1_ = 50; the “loss” penalty *C*_2_ = 30. Optimal labels are in red.

The next simplest tree topology—three-leaf trees—is presented in Figure 2. There are two nodes to label, the inner node and the root.

- Figure 2(a): the inner node is labeled analogically to the root in Figure 1(c): any *k*  125 ≤ *k* ≤ 136 is equally good to label the inner node; the root node is labeled analogically to the root in Figure 1(b): (*Z*(root) = *C*_1_ + (136 − 125)) > (*X*(root) = *C*_2_ + 11) → the root is labeled by any *k*  125 ≤ *k* ≤ 136, that is, by 125.
- Figure 2(b): labeling is similar to that of Figure 1(a).
- Figure 2(c): the inner node is labeled analogically to Figure 2(a): any label 125 ≤ *k* ≤ 136 is good to label it; the score is equal to 136 − 125 = 11. The root should be labeled by 136 because 125 < 136 < 141.

**Figure 3:**
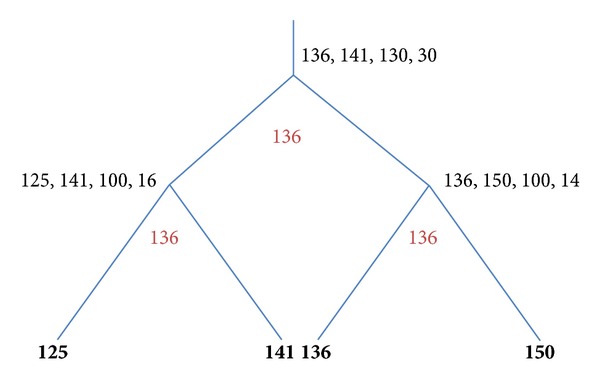
Assignment of bottom-up stage values (left, right, *Z*, and *X*) in a 4-leaf tree with all four leaves labeled by positive integers. The “gain” penalty *C*_1_ = 50; the “loss” penalty *C*_2_ = 30. Optimal labels are in red.

Determination of the optimal labeling of the four-leaf trees is very similar to the examples described above.  Figure 3 illustrates labeling of the tree where all four leaves have nonzero labels: ((125, 141), (136, 150)). Labeling of the inner nodes is as above (Figure 2(c)): [125, 141] and [136, 150]. All integers of the intersection between these two close intervals are optimal values to label the root: [125, 141]∩[136, 150] = [136, 141]. In Figure 3 we present the value 136 as a chosen suitable label.

**Figure 4:**
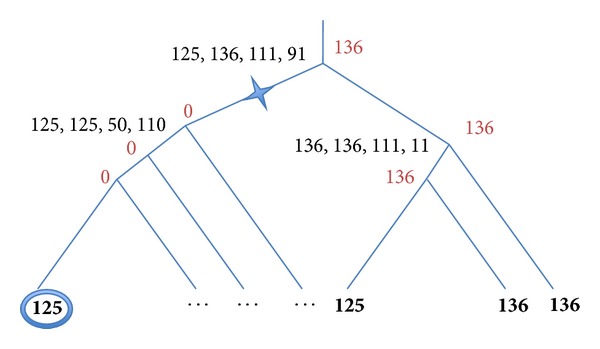
Labeling of a “peculiar” tree. The left subtree has three zero and one nonzero leaf, while the right subtree has three nonzero leaves. The “gain” penalty *C*_1_ = 50; the "loss" penalty *C*_2_ = 30. Optimal labels are in red.

**Figure 5:**
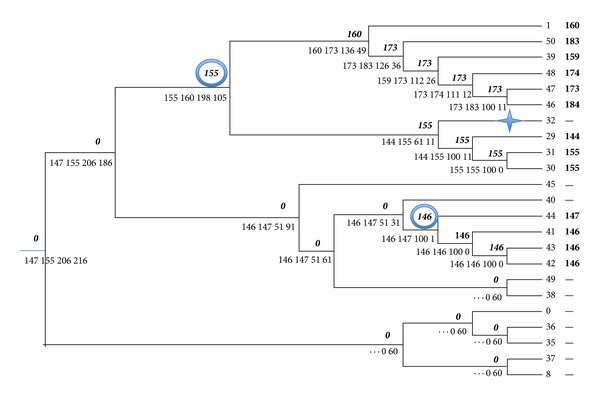
Archaeal part of Figure 4(b) from [7] labeled accordingly to COG0835.

Examples of the trees with very distinct subtrees are presented in Figures 4 and 5. In Figure 4 we present a tree obtained by merging two very different subtrees. The left 4-leaf subtree has very obvious intuitive labeling of internal nodes: all nodes should be labeled by zero. The right subtree is identical to the tree presented in Figure 2(c). Merging of these two subtrees produces bottom-up stage values (left, right, *Z*, and *X*) to the new root equal to [125, 136, 111, 91]. In spite of assignins the interval [125, 136] to the root only the value 136 provides the optimal solution. (We would like to express our gratitude to the anonymous reviewer for bringing our attention to this situation.) We formulate this rule below describing traceback stage of the algorithm. Figure 4 is chosen to illustrate labeling of nodes similar to the root of the tree.

After considering these few simple examples, we describe the algorithm.

#### 3.2.2. Bottom-Up Stage

*Initiation.* Given labeling of the leaf nodes of *G*(*p*_1_, …, *p*_*n*_) at the tips of the tree the *σ*_*i*_(*j*) are easy to compute. The cost is 0 if the observed integer *p*_*i*_ is integer *j*, and

(13) $$\begin{array}{c} C_{1}+C_{2}\;\text{otherwise}. \end{array}$$

*Iteration.* Doing a postorder tree traversal assign successively to each node in the tree the abovementioned four values left(*a*), right(*a*), *Z*(*a*), and *X*(*a*). An interval [left(*a*), right(*a*)] is assigned according to the following rule: if anyone of two children intervals is not defined, then assign the interval of the other child; otherwise, a parent interval is either an intersection of the intervals of its children or an interval that lies between these intervals if their intersection is empty. *Z* is a cost value *σ*_*a*_(0), where for the Manhattan distance we can rewrite (10) as

(14) Z(a)=σa(0)=min⁡⁡(σl(0),C1+σl(j))+min⁡⁡(σr(0),C1+σr(j)),X(a)=σa(i)=min⁡⁡(min⁡j⁡[θ(i,j)+σl(j)],C2+σl(0))+min⁡⁡(min⁡k⁡[θ(i,k)+σr(k)],C2+σr(0))=min⁡(min⁡j⁡[|i−j|+σl(j)],C2+σl(0))+min⁡⁡(min⁡j⁡[|i−j|+σr(j)],C2+σr(0)).

#### 3.2.3. Pseudocode

For more details see Pseudocode 1.

#### 3.2.4. Traceback Stage

*Interval Correction Rule.* Following the bottom-up stage four values left(*a*), right(*a*), *Z*(*a*), and *X*(*a*) are assigned to every internal node *a* of the tree. An interval (left(*a*), right(*a*)) should be diminished if one of the edges connecting the node *a* with its son becomes of type (*k*, 0), *k* > 0. Let us denote sons of the node *a* by *l*(*a*) and *r*(*a*). Correction condition Ω(*a*) would be formulated as

(15) Ω(a)=(X(a)≤Z(a)) and   ((X(l(a))>Z(l(a)))V(X(r(a))>Z(r(a)))).

If Ω(*a*) is TRUE, then the bounds of the corrected interval would be obtained by intersection of the interval associated with the node with the corrected interval associated with the corresponding son:

(16) if  X(l(a))>Z(l(a)),then[left'(a),right'(a)]=[left(a),right(a)]  ∩[left(r(a)),right(r(a))]else[left'(a),right'(a)]=[left(a),right(a)]  ∩[left(l(a)),right(l(a))];

Otherwise, the bounds of the corrected interval would not be changed from the original ones:

(17) [left'(a),right'(a)]=[left(a),right(a)].

*Initiation.* Labeling of the root: if *X*(*N*) ≤ *Z*(*N*), then correct the root interval according to (15)–(17), and then choose an integer from the corrected interval assigned to the root node otherwise choose 0—it is the root label *π*(*N*).

*Iteration.* Doing a preorder tree traversal, successively label each node in the tree either by an integer from the corrected interval assigned to this node which is the nearest to its parent label (it may be either the value equal to the parent label or the boundary value of the interval assigned with the node) or by 0.

The proof of the correctness of a simpler algorithm (without zero-labeled leaves) is published in [15]. In the Appendix there are several lemmas, from which the correctness of the algorithm presented here results.

#### 3.2.5. Example

In Figure 5 of [7] the consensus trees obtained from 100 genome trees were presented. The trees were produced on the basis of 80% randomly chosen COGs, and the right tree was produced on the basis of 15%-jackknifing (the explanations in the text of [7]). This tree possesses phylogenetic reasonableness.

- The representatives of both prokaryotic Kingdoms: Eubacteria and Archaea are clustered separately. In other words, Archaeal organisms (genomes 0, 1, 8, 29–32, and 35–50) form a monophyletic group.
- Euryarchaeota and Crenarchaeota form monophyletic groups.

A part of this tree was selected to illustrate the algorithm. We took the upper part of the tree related exclusively to Archaea (see A/B marked arrow in Figure 4(b) from [7]) and placed the root at the point dividing all Archaeal genomes into Euryarchaeota and Crenarchaeota (see E/C marked arrow in Figure 4(b) from [7]). Thus, Figure 5 is a part of Figure 4(b) from [7] labeled according to COG0835. This COG was randomly selected as suitable for purposes of illustration.  Table 1 presents a list of Archaeal genomes from the whole set of genomes that were used for a genome tree construction (Figure 4(b) from [7]).  Table 2 presents the lengths of the Archaeal proteins of this COG.

To assign labels to the leaves of the tree of Figure 5 two preprocessing steps were done: (1) taking off outliers, the lengths 328 of the *H*. *marismortui* protein and 344 of the *M. hungatei* protein are obvious outliers; (2) taking the median value of paralog's lengths of the genomes 30, 31, 46, 48, and 50. Figure 5 presents results of application of the bottom-up and traceback stages of the algorithm to this tree: a quartet that was assigned to a node *a* at the bottom upstage is shown under the edge linking the node *a* and its parent node, a label that was assigned to the node *a* at the traceback stage, is shown over the same edge.

As we can see the root is labeled by zero. There are two gene-birth events and one gene-death event. One gene was born with the length of 155 and another gene birth is labeled by 146. Genome number 32 (*Haloquadratum walsbyi*) has no protein from COG0835, while other *Haloarchaea* (genomes 29–31) do have. Thus, the edge connecting with leaf labeled by 32 is marked with a gene-loss symbol.

## 4. Discussion

In [15] the algorithms to find the optimal labeling of the vertices of the tree under Wagner parsimony were presented. A simple extension of the problem could be finding the optimal labeling of the vertices of the tree with nonnegative integers. This more realistic approach requests special consideration of zero labeling. Wedges of type (*k*, 0), *k* > 0, should be scored differently from wedges of type (0, *k*), *k* > 0, because the (*k*, 0) notes gene loss, while (0, *k*) notes gene gain. These events should be scored differently. Interestingly, this differentiated scoring in addition to tree labeling resulted in reconstruction of “parsimonious” evolutionary scenario. Reconstruction of a gene evolution along a species tree is an interesting and principal problem. Lyubetsky and his coworkers contributed a lot to formulation and solving this problem. In their studies [16–22] the authors tackled mainly two important and sophisticated phylogenetic problems. The obtained results are partially reviewed in the first section of [22] which also provides an extended biological background and relevant references. Reconstruction of a gene evolution along a species tree (to build the evolutionary scenario), following the approach of Lyubetsky et al., is to find an optimal mapping of a gene tree into a species tree. (An example of a different approach was presented in [14].) The second problem is to construct a supertree from the given set of gene trees.

As it was mentioned in [22], the first problem, stated as a tree-into-tree mapping, is solved in polynomial (often linear, and at maximum cubic) time even for the case of time slices and horizontal gene transfers. The algorithms presented in our study are polynomial as well.

Choosing *C*_1_ (a gain penalty), *C*_2_ (a loss penalty), and *θ* (a label change penalty) is crucial for reconstruction of trustworthy evolutionary scenario. However, it is very difficult task and we cannot claim categorically that choosing “correct” parameters of the model will result in truly reliable reconstruction. We do plan to make a comparison between results obtained by abovementioned methods of Lyubetsky and ours (work in progress).

To prepare input for the algorithm, as it was done above for 3.2.5, the original data is to be transformed to the following format: to each (genome, COG) pair one standardized protein length should be assigned (as we described in [7]). For a given COG, each organism is represented by a calculated length—a median length of all paralogous proteins. A natural extension would be to formulate the labeling problem taking into account existence of paralogs.

We may define a *k*-*tuple* integer labeling Π of *G* as a mapping Π from *G* to a set of *k*-tuples composed of integers Π(*v*) = {*π*_1_(*v*), *π*_2_(*v*),…, *π*_*k*(*v*)_(*v*)}, where *π*_*i*_(*v*) ≤ *π*_*i*+1_(*v*) for all 1 ≤ *i* < *k*(*v*). The simplest extension would be to introduce the case with *identical* sizes of *k*-tuples composed of *nonnegative* integers. A *uniform* *k*-tuple integer labeling Π_*c*_ of *G* is characterized by a constant *k*(*v*) for all *v*. The stretch of the edge *vw* in a Π_*c*_(*G*) is a simple sum *c*_*vw*_ = ∑_*i*=1_^*k*^ *φ*(*π*_*i*_(*v*), *π*_*i*_(*w*)) · *φ*(*x*, *y*) is defined as in (1). Given a uniform *k*-tuple integer labeling of the leaves of G the minimum sum problem is to find a labeling which minimizes the total sum of the stretches of the edges. Some ***π***_**i**_(**v**) = 0. The minimum sum problem is that of minimizing **s**(**G**) = ∑_∀{*vw*}∈*E*(*G*)_ *c*_*vw*_ over all Π_*c*_ for given *k*. By some modifications of the algorithms presented in this paper the minimizing *k*-tuple labeling can be found. This model again is a gain-loss model. More sophisticated extension must provide more realistic definition of distance between two *k*-tuples composed of positive integers by introducing duplication events.

## Figures and Tables

**Table 1:** List of archaeal genomes for Figure 4.

No.	Name	Kingdom	Group
0	*Aeropyrum pernix* K1	A	C
1	*Archaeoglobus fulgidus* DSM 4304	A	E
8	*Caldivirga maquilingensis* IC-167	A	C
29	*Haloarcula marismortui* ATCC 43049	A	E
30	*Halobacterium salinarum* R1	A	E
31	*Halobacterium *sp. NRC-1	A	E
32	*Haloquadratum walsbyi* DSM 16790	A	E
35	*Hyperthermus butylicus* DSM 5456	A	C
36	*Ignicoccus hospitalis* KIN4/I	A	C
37	*Metallosphaera sedula* DSM 5348	A	C
38	*Methanobrevibacter smithii* ATCC 35061	A	E
39	*Methanococcoides burtonii* DSM 6242	A	E
40	*Methanococcus aeolicus* Nankai-3	A	E
41	*Methanococcus maripaludis* C5	A	E
42	*Methanococcus maripaludis* C6	A	E
43	*Methanococcus maripaludis* C7	A	E
44	*Methanococcus maripaludis* S2	A	E
45	*Methanosaeta thermophila* PT	A	E
46	*Methanosarcina acetivorans* C2A	A	E
47	*Methanosarcina barkeri* str. fusaro	A	E
48	*Methanosarcina mazei *Go1	A	E
49	*Methanosphaera stadtmanae *DSM 3091	A	E
50	*Methanospirillum hungatei* JF-1	A	E

Notations of the groups: E: *Euryarchaeota*, C: *Crenarchaeota*.

**Table 2:** Protein lengths of the chemotaxis signal transduction proteins. Archaeal part of COG0835.

Number	COG	Length	Genome name
1	835	160	*Archaeoglobus fulgidus* DSM 4304
29	835	144	*Haloarcula marismortui* ATCC 43049
29	835	328	*Haloarcula marismortui *ATCC 43049
30	835	132	*Halobacterium salinarum *R1
30	835	178	*Halobacterium salinarum* R1
31	835	132	*Halobacterium* sp. NRC-1
31	835	178	*Halobacterium* sp. NRC-1
39	835	159	*Methanococcoides burtonii *DSM 6242
41	835	146	*Methanococcus maripaludis* C5
42	835	146	*Methanococcus maripaludis* C6
43	835	146	*Methanococcus maripaludis *C7
44	835	147	*Methanococcus maripaludis* S2
46	835	182	*Methanosarcina acetivorans* C2A
46	835	184	*Methanosarcina acetivorans* C2A
47	835	173	*Methanosarcina barkeri* str. fusaro
48	835	159	*Methanosarcina mazei *Go1
48	835	189	*Methanosarcina mazei *Go1
50	835	124	*Methanospirillum hungatei *JF-1
50	835	167	*Methanospirillum hungatei *JF-1
50	835	169	*Methanospirillumhungatei* JF-1
50	835	169	*Methanospirillum hungatei* JF-1
50	835	174	*Methanospirillumhungatei* JF-1
50	835	176	*Methanospirillum hungatei *JF-1
50	835	183	*Methanospirillum hungatei *JF-1
50	835	187	*Methanospirillum hungatei* JF-1
50	835	189	*Methanospirillum hungatei *JF-1
50	835	190	*Methanospirillum hungatei* JF-1
50	835	198	*Methanospirillum hungatei* JF-1
50	835	200	*Methanospirillum hungatei* JF-1
50	835	344	*Methanospirillum hungatei *JF-1
50	835	779	*Methanospirillum hungatei *JF-1

## Appendix

Lemma A.1 (root optimal label) Suppose that the root node is called *rt* and suppose that its children are called *l* and *r*. The claim is

1. if (*π*(*l*) = *π*(*r*) = 0), then *π*(*rt*) = 0 else
2. if (*π*(*l*) = 0), then *π*(*rt*) = *π*(*r*) else
3. if (*π*(*r*) = 0), then *π*(*rt*) = *π*(*l*) else
4. if (*π*(*l*) ≤ *π*(*r*)), then *π*(*l*) ≤ *π*(*rt*) ≤ *π*(*r*) else
5. *π*(*l*) ≥ *π*(*rt*) ≥ *π*(*r*).

Proof If we consider a subtree with the node *k* as a root then *σ*_*k*_(*i*) designates the minimal cost, given that node *k* has a label *i*:

(A.1) S(G)=min⁡i⁡σN(i)=min⁡i⁡[φ(π(rt),π(l))   +φ(π(rt),π(r))+σl(π(l))+σr(π(r))].

Proof of the subclaims (1)–(3) is trivial. Case (4) is

(A.2) S(G)=min⁡i⁡σN(i)=min⁡i[|π(rt)−π(l)|+|π(rt)−π(r)|   +σl(π(l))+σr(π(r))].

Let us introduce a new numbering *π*′ by changing only the root label: *π*′(*rt*) = *π*(*l*). It is easy to see that *S*_*π*′_(*G*) < *S*_*π*_(*G*). It means that for optimal integer labeling *π* the following is correct: *π*(*rt*) ≥ *π*(*l*). Likewise, we prove that for optimal integer labeling *π*(*rt*) ≤ *π*(*r*). Let us denote *k* = *π*(*rt*) : *π*(*l*) ≤ *k* ≤ *π*(*r*):

(A.3) ∀k(π(l)≤k≤π(r))Sπ(G)  =k−π(l)+Sπ(l)+π(r)−k+Sπ(r),  q.e.d.

Lemma A.2 (leaf parent optimal interval) Every node of the optimal integer labeling that all its children are leaf nodes has either a zero label or a label between labels of its children. Suppose that the root node is called a and suppose that its children are called *l* and *r*. The claim is

1. if (*π*(*l*) = *π*(*r*) = 0), then *π*(*a*) = 0 else
2. if (*π*(*l*) = 0), then *π*(*a*) = *π*(*r*) else
3. if (*π*(*r*) = 0), then *π*(*a*) = *π*(*l*) else
4. if (*π*(*l*) ≤ *π*(*r*)), then *π*(*l*) ≤ *π*(*a*) ≤ *π*(*r*) else
5. *π*(*l*) ≥ *π*(*a*) ≥ *π*(*r*).

Proof
Figure 1 illustrates this lemma. Proof of the subclaims (1)–(3) is trivial. In case of condition (4) let us prove that for optimal integer labeling *π*(*a*) ≥ *π*(*l*). Suppose *π*(*a*) < *π*(*l*). Let us denote (*π*(*l*) − *π*(*a*)) = *δ*; *π*(*r*) − *π*(*l*) = *γ*. Let us introduce a new numbering *π*′ by changing only the label of the node *a*: *π*′(*a*) = *π*(*l*). It is to show that *S*_*π*′_(*G*) < *S*_*π*_(*G*). Indeed,

(A.4) Sπ′(G)=Sπ(G)−|π(k)−π(j)|−(pi−π(k))−(pi+1−π(k))+|π′(k)−π(j)|+(pi−π′(k))+(pi+1−π′(k))=Sπ(G)+(|pi−δ−π(j)|−|pi−π(j)|)−(pi−π(k))−(pi+1−π(k))+(pi−pi)+(pi+1−pi)=Sπ(G)+(|pi−δ−π(j)|−|pi−π(j)|)−δ−(δ+γ)−γ=Sπ(G)−δ.

Likewise, we prove that for optimal integer labeling *π*(*k*) ≤ *p*_*i*+1_. Q.e.d. *p*_*i*_ ≤ *π*(*k*) ≤ *p*_*i*+1_.

Lemma A.3 (parent optimal interval—(I)) An optimal label of a parent either is equal to zero or lies between extreme values of optimal labels of its children. If an optimal integer labeling *π* provides the labels of two siblings *i*_1_ and *i*_2_ satisfying the conditions *a*_1_ ≤ *π*(*i*_1_) ≤ *b*_1_ & *a*_2_ ≤ *π*(*i*_2_) ≤ *b*_2_, then the label of their parent *k* satisfies min⁡(*a*_1_, *b*_1_, *a*_2_, *b*_2_) ≤ *π*(*k*) ≤ max⁡(*a*_1_, *b*_1_, *a*_2_, *b*_2_). Proof is as for Lemma A.1.

Lemma A.4 (parent optimal interval—(II)) An optimal label of a parent in case of the empty intersection of the optimal intervals of its children lies between these intervals. If an optimal integer labeling *π* provides the labels of two siblings *i*_1_ and *i*_2_ satisfying the conditions (*a*_1_ ≤ *π*(*i*_1_) ≤ *b*_1_) & (*a*_2_ ≤ *π*(*i*_2_) ≤ *b*_2_) then if (*b*_1_ ≤ *a*_2_) then *b*_1_ ≤ *π*(*k*) ≤ *a*_2_ else if (*b*_2_ ≤ *a*_1_) then *b*_2_ ≤ *π*(*k*) ≤ *a*_1_.

Proof (1) *b*_1_ ≤ *a*_2_. Let us assume *π*(*k*) < *b*_1_; *π*(*k*) = *b*_1_ − *α*. Then we introduce a new labeling *π*′ by changing labels for three nodes: *π*′(*k*) = *b*_1_; *π*′(*i*_1_) = *b*_1_; *π*′(*i*_2_) = *a*_2_:

(A.5) Sπ(G)−Sπ′(G) =(|π(j)−π(k)|−|π(j)−π′(k)|)  +(|π(k)−π(i1)|−|π′(k)−π′(i1)|)  +(|π(k)−π(i2)|−|π′(k)−π′(i2)|),(|π(j)−π(k)|−|π(j)−π′(k)|) =|π(j)−b1+α|−|π(j)−b1|=α,(|π(k)−π(i1)|−|π′(k)−π′(i1)|) =((π(k)−π(i1))−(π′(k)−π′(i1))) =b1−α−π(i1)−b1+b1 =b1−α−π(i1),(|π(k)−π(i2)|−|π′(k)−π′(i2)|) =π(i2)−b1+α−a2+b1,Sπ(G)−Sπ′(G)=α+b1−α−π(i1)        +π(i2)+α−a2=(b1−π(i1))        +α+(π(i2)−a2)>0.

From assumption *π*(*k*) < *b*_1_ follows that *π* is not an optimal labeling. Similarly, we can prove that from assumption *π*(*k*) > *a*_2_ follows that *π* is not an optimal labeling. (2) *b*_2_ ≤ *a*_1_. Similarly to (1) let us assume *π*(*k*) < *b*_2_; *π*(*k*) = *b*_2_ − *α*. Then we introduce a new labeling *π*′ by changing labels for three nodes: *π*′(*k*) = *b*_2_; *π*′(*i*_1_) = *a*_1_; *π*′(*i*_2_) = *b*_2_. *S*_*π*_(*G*) − *S*_*π*′_(*G*) > 0, so we have a contradiction with the statement that *π*(*G*) is an optimal labeling.

Lemma A.5 (parent optimal interval—(III)) An optimal interval of a parent is either an intersection of the optimal intervals of its children or an interval that lies between these intervals in case that their intersection is empty. If an optimal integer labeling *π* provides the labels of two siblings *i*_1_ and *i*_2_ satisfying the conditions *a*_1_ ≤ *π*(*i*_1_) ≤ *b*_1_  &  *a*_2_ ≤ *π*(*i*_2_) ≤ *b*_2_, then the label of their parent *k* satisfies the following condition:

if ([*a*_1_, *b*_1_]∩[*a*_2_, *b*_2_]) ≠ *∅* then *π*(*k*) ∈ [*a*_1_, *b*_1_]∩[*a*_2_, *b*_2_]) else
if *b*_1_ < *a*_2_  then *π*(*k*) ∈ [*b*_1_, *a*_2_] else *π*(*k*) ∈ [*b*_2_, *a*_1_].

For example, if *a*_1_ ≤ *a*_2_ ≤ *b*_1_ ≤ *b*_2_, then Lemma A.5 states that *π*(*k*) satisfies the following condition *a*_2_ ≤ *π*(*k*) ≤ *b*_1_. Proof is similar to proof in Lemma A.4.
